# Vitamin D Supplementation in Critically Ill Patients: A Meta-Analysis of Randomized Controlled Trials

**DOI:** 10.7759/cureus.24625

**Published:** 2022-04-30

**Authors:** Sahib Singh, Sauradeep Sarkar, Kushagra Gupta, Amit Rout

**Affiliations:** 1 Internal Medicine, Sinai Hospital of Baltimore, Baltimore, USA; 2 Pulmonary Critical Care Medicine, East Carolina University, Greenville, USA

**Keywords:** randomized controlled trials, intensive care unit, critically ill patients, cholecalciferol, vitamin d

## Abstract

Randomized controlled trials (RCTs) have reported conflicting outcomes with the use of vitamin D in critically ill patients. With reporting of newer RCTs, we conducted this updated meta-analysis. Electronic databases were searched for RCTs comparing vitamin D with placebo in critically ill patients admitted to the intensive care unit (ICU). A random-effects meta-analysis was used to calculate the risk ratio (RR) and standardized mean difference (SMD) with a 95% confidence interval (CI). Eleven RCTs with a total of 2,187 patients (vitamin D: n = 1,120; placebo: n = 1,067) were included. Vitamin D when compared to placebo was associated with the decreased duration of mechanical ventilation (SMD = -0.50; 95% CI = [-0.97, -0.03]; p = 0.04) and ICU stay (SMD = -0.60; 95% CI = [-1.03, -0.16]; p = 0.007) without any difference in the mortality (RR = 0.85; 95% CI = [0.68, 1.04]; p = 0.12) and length of hospital stay (SMD = -0.21; 95% CI = (-0.51, 0.09); p = 0.18]. Subgroup analysis showed that parenteral vitamin D may reduce the risk of mortality (RR = 0.54; 95% CI = [0.35, 0.83], p = 0.005). Vitamin D supplementation in critically ill patients decreases the duration of mechanical ventilation and ICU stay. Further studies should identify specific groups of patients who will derive the most benefit from vitamin D supplementation.

## Introduction and background

Vitamin D deficiency is commonly noted in critically ill patients and has been shown to be associated with worse clinical outcomes. Studies have shown that vitamin D deficiency is associated with increased risk for respiratory infections, increased length of stay, and mortality [1-3]. Apart from its role in bone and calcium metabolism, vitamin D works as an immuno-modulator, decreases inflammatory cytokines, and may have a role in lung protection [4,5]. Vitamin D supplementation has been shown to be beneficial in many non-critical care settings like colon cancer and inflammatory bowel disease [6-8].

Randomized controlled trials (RCTs) assessing the role of vitamin D in critically ill patients had shown conflicting results [9-12]. Some trials suggested possible benefits in mortality, while others suggested decreased intensive care unit (ICU) stay. Current guidelines do not suggest routine measurement of vitamin D levels or its supplementation in critically ill patients. Previous meta-analyses have also shown variable results regarding the role of vitamin D supplementation; some of the major limitations of those meta-analyses were smaller study sample sizes and a limited number of included studies [13-15]. One of the recently published and the largest RCT till date, the VIOLET trial, showed a numerical increase in the mortality in patients receiving vitamin D supplementation compared to placebo [16]. With the reporting of newer RCTs and concern for worse outcomes with vitamin D supplementation, we performed this updated meta-analysis to evaluate the role of vitamin D in critically ill patients.

## Review

Materials and methods

Search Strategy

We searched MEDLINE, Embase, and Cochrane databases for all the RCTs published until October 31, 2020. We used search terms like “Vitamin D,” “cholecalciferol,” “critically ill patients,” and “intensive care unit” in different combinations. Studies that involve RCTs of adult human subjects reporting clinical outcomes in critically ill/ICU patients who were treated with vitamin D versus a placebo arm and those that report at least one clinical endpoint based on the treatment approach were included. The main exclusion criteria were non-randomized study designs.

Data Collection

Two reviewers (SS and SS) independently screened the study reports for eligibility, assessed the risk of bias, and collected data from each eligible study. Any differences between the two reviewers were resolved with consensus after discussion with the third reviewer (AR). From the eligible RCTs, data on study characteristics like study design, year of publication, inclusion and exclusion criteria, sample size, follow-up period, baseline patient characteristics, treatment data, and clinical outcomes at the longest available follow-up were obtained. Subgroups were made based on the route of vitamin D administration: enteral or parenteral route. The outcomes of interest were mortality, duration of mechanical ventilation, ICU stay, and hospital stay.

Study Analysis

This meta-analysis was conducted following the Preferred Reporting Items for Systematic Reviews and Meta-Analyses (PRISMA) guidelines [17]. We used Cochrane Review Manager, version 5.4 (Cochrane, London, United Kingdom), for study analysis [18]. Mean and standard deviations were extrapolated from median and range using the statistical method outlined by Hozo et al. [19]. For dichotomous clinical data, pooled risk ratio (RR) and 95% confidence interval (CI) were calculated using the random-effects models with the Mantel-Haenszel method. For continuous variables, we computed standardized mean difference (SMD) with 95% CI using the inverse variance method. A p-value of 0.05 or less was considered statistically significant. Study heterogeneity was assessed by calculating I-squared statistic; heterogeneity was considered significant in the case of I^2^ > 50%. Sensitivity analysis was performed by excluding each trial from the final analysis. Forest plots were generated to demonstrate the relative effect size of vitamin D supplementation versus placebo for individual clinical endpoints.

Results

**Figure 1:**
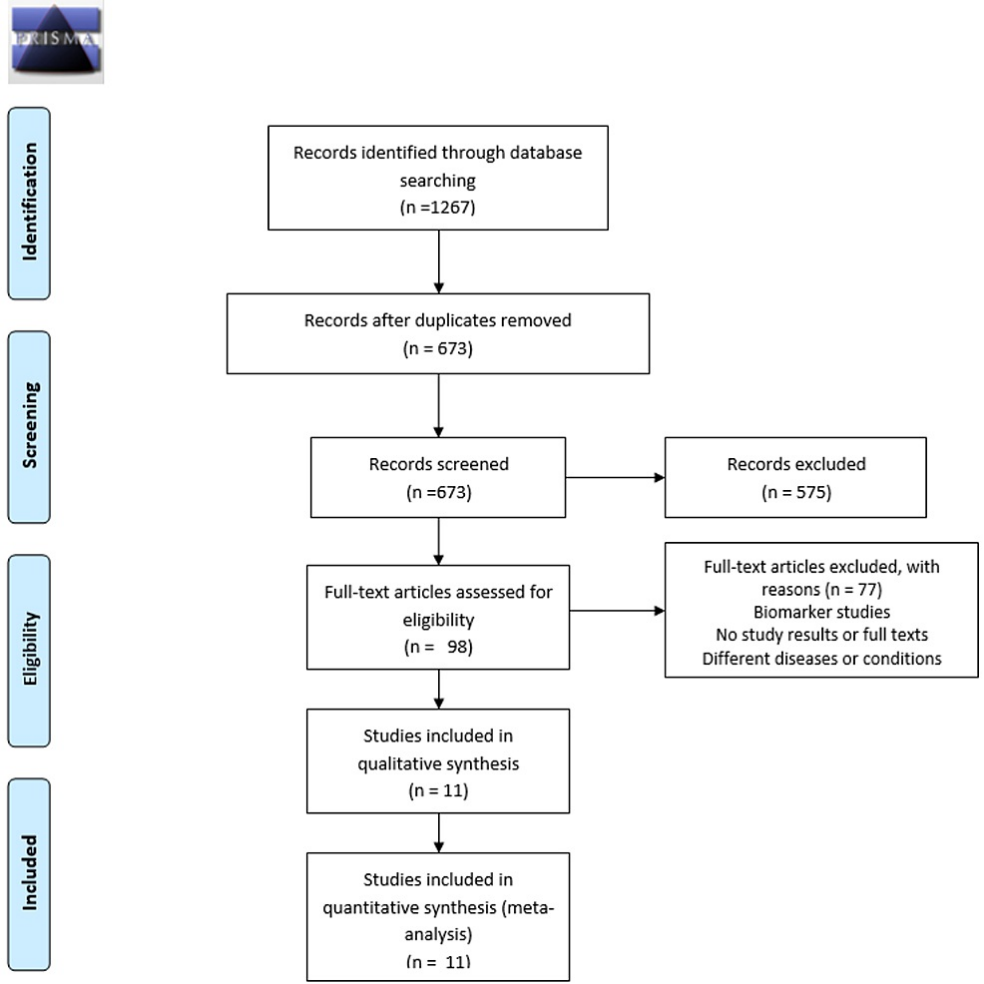
PRISMA flow diagram PRISMA: Preferred Reporting Items for Systematic Reviews and Meta-Analyses. Source: References [9-12,16,20-25].

The initial search yielded 1,267 studies out of which 11 RCTs were identified [9-12,16,20-25]. Figure 1 shows the PRISMA flow diagram for search strategy. Data for one of the trials was accessed using an abstract [22]. A total of 2,187 patients with 1,120 in the vitamin D arm and 1,067 in the placebo arm were included in the final analysis. The mean duration of follow-up was two months for all the trials except one which was until hospital/ICU stay [23]. The mean age of the study population was 58 years, and 61% of the study population were men. Vitamin D formulation was enteral (oral or via feeding tube) in six studies; parenteral (intravenous or intramuscular) in four studies, and both in one study. Baseline vitamin D levels were assessed in six studies. Among the 11 studies, six studies included patients admitted with any medical cause requiring ICU level of care, three studies included sepsis/septic shock patients, one study with only neuro-critical care patients, and one study included patients exclusively with ventilator-associated pneumonia. Table 1 shows the important characteristics of included trials.

**Table 1:** Characteristics of the studies included in the meta-analysis RECTIFY: RandomizEd Clinical TrIal oF hYpovitaminosis D; VIOLET: Early high-dose vitamin D3 for critically ill, vitamin D-deficient patients; VITdAL-ICU: Effect of high-dose vitamin D3 on hospital length of stay in critically ill patients with vitamin D deficiency; Vit D: Vitamin D group; ICU: Intensive care unit; 25(OH)D: 25-Hydroxyvitamin D level; NA: Not available; PO: Per-oral; IU: International units; IV: Intravenous; IM: Intramuscular; Inj: Injection. Source: References [9-12,16,20-25].

Study (year)	Total N (Vit D/Control)	Age (years) (Vit D/Control)	Male (%) (Vit D/Control)	Follow-up	Major inclusion criteria	Vitamin D dosing/formulation
Amrein et al. (2011) [10]	12/13	61.1/64.1	75/77	28 days	ICU stay > 48 hours & 25(OH)D level ≤ 20 ng/ml	PO 540,000 IU of vitamin D3
Leaf et al. (2014) [21]	36/31	68/58	61/48	28 days	Severe sepsis or septic shock	IV calcitriol 2 mcg
Amrein et al. (2014) [9]	237/238	63.9/65.3	65/65.1	180 days	ICU stay > 48 hours & 25(OH)D < 20 ng/ml	PO loading dose of 540,000 IU of vitamin D3; then 90,000 IU/month x 5 months of oral vitamin D3
Quraishi et al. (2015) [12]	20/10	63/65	60/60	30 days	ICU admission for new-onset sepsis	PO 200,000 or 400,000 IU vitamin D3
Han et al. (2016) [11]	20/10	56.4 & 68.1/64.8	55.6 & 72.7/60	84 days	Admitted to ICU & expected to be on mechanical ventilation for at least 72 hours	PO 250,000/500,000 IU vitamin D3
Miroliaee et al. (2017) [20]	24/22	57.8/56.5	66.6/59.1	28 days	Diagnosed with ventilator-associated pneumonia	IM Vitamin D 300,000 units
Ding et al. (2017) [22]	29/28	NA	NA	28 days	Sepsis, severe sepsis, & 25(OH)D ≤ 30 μg/L	Inj IM 300,000 IU vitamin D3
Karsy et al. (2019) [25]	134/133	52.9/55.1	58.2/55.6	30 days	Admission to the neuro-critical care, expected ICU stay ≥ 48 hours & 25(OH)D ≤ 20 ng/ml	PO 540,000 IU of vitamin D3
Hasanloei et al. (2019) [23]	48/24	50 & 44.4/48.7	70.8 & 45.8/50	Till hospital stay	Mechanical ventilation ≥ 48 hours and ICU stay ≥ 7 days & 25(OH)D 10-30 ng/ml	PO 50,000 IU daily for 6 days & IM 300,000 IU cholecalciferol
Miri et al. (2019) [24]	22/18	52/56	63.6/72.2	28 days	On mechanical ventilation	IM vitamin D 300,000 IU
Ginde et al. (2019) [16]	538/540	56.5/54.6	57.4/55.9	90 days	Risk factors for ICU admission & 25(OH)D < 20 ng/ml	PO 540,000 IU of vitamin D3

**Figure 2:**
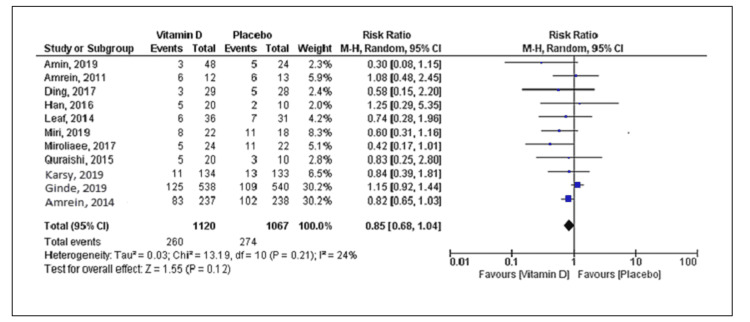
Forest plot showing the risk ratio for mortality Source: References [9-12,16,20-25].

**Figure 3:**
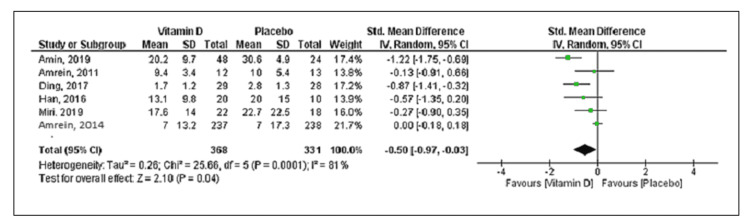
Forest plot showing the standard mean difference for the duration of mechanical ventilation Source: References [9-12,16,20-25].

**Figure 4:**
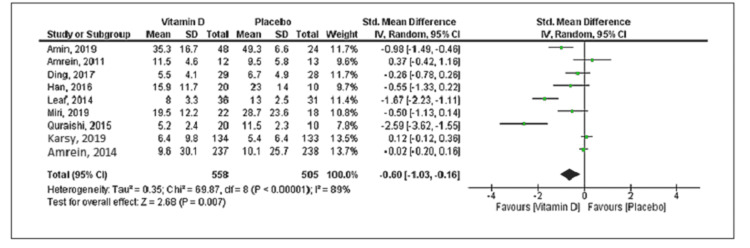
Forest plot showing the standard mean difference for the ICU stay Source: References [9-12,16,20-25].

**Figure 5:**
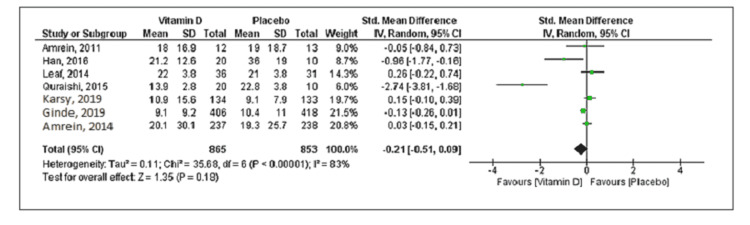
Forest plot showing the standard mean difference for the hospital stay Source: References [9-12,16,20-25].

Eleven studies reported mortality with a total of 534 deaths (vitamin D group: n = 260 and placebo group: n = 274) out of the 2,187 patients. Compared to placebo, vitamin D supplementation was associated with a decrease in mortality, but the difference was statistically not significant (RR = 0.85; 95% CI = [0.68, 1.04]; p = 0.12) (Figure 2). No significant heterogeneity was noted in the analysis (I^2 ^= 24%). Six trials reported the duration of mechanical ventilation, and nine trials reported the length of ICU stay. Vitamin D supplementation was associated with decreased duration of mechanical ventilation (SMD = -0.50; 95% CI = [-0.97, -0.03]; p = 0.04) and length of ICU stay (SMD = -0.60; 95% CI = [-1.03, -0.16]; p = 0.007) when compared to placebo (Figures 3, 4). The results were associated with significant heterogeneity of I^2 ^= 81% for mechanical ventilation and I^2 ^= 89% for ICU stay. Seven trials reported the length of hospital stay. Compared to placebo, vitamin D was associated with a similar length of hospital stay (SMD = -0.21; 95% CI = [-0.51, 0.09]; p = 0.18) (Figure 5). The results were associated with significant heterogeneity of I^2 ^= 83%.

**Figure 6:**
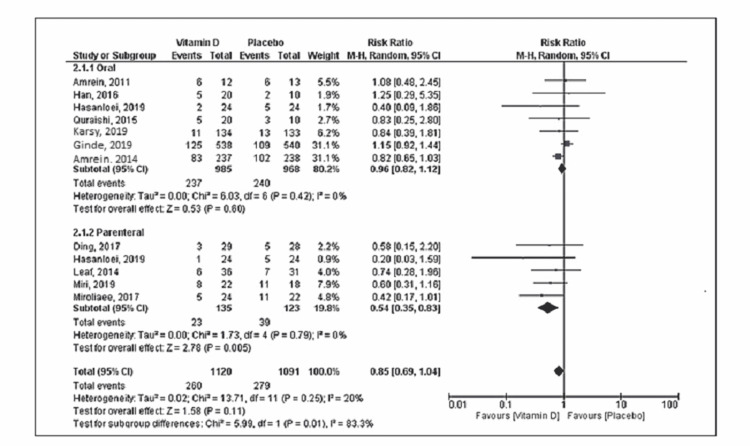
Subgroup analysis forest plot for mortality Source: References [9-12,16,20-25].

**Figure 7:**
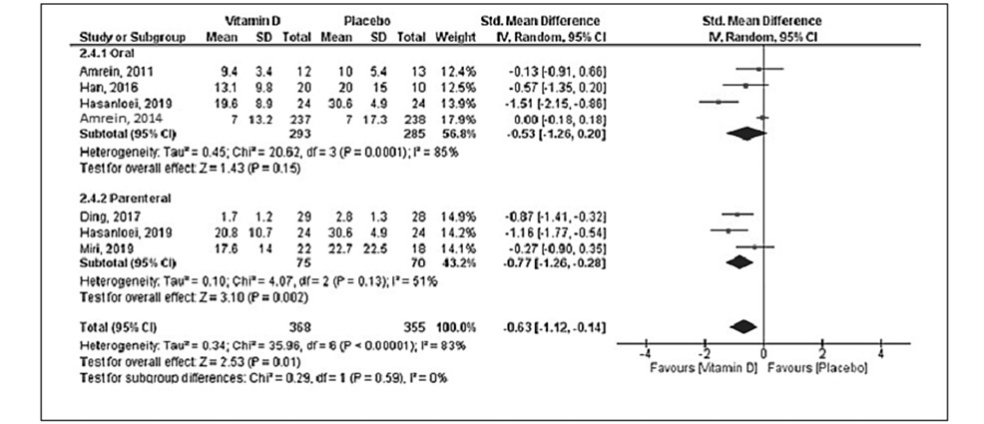
Subgroup analysis forest plot for the duration of mechanical ventilation Source: References [9-12,16,20-25].

**Figure 8:**
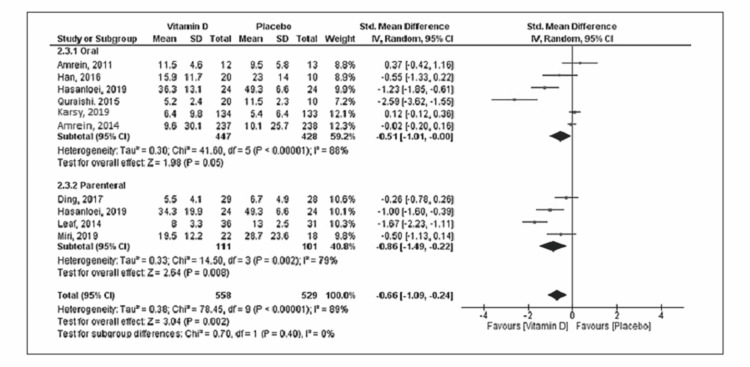
Subgroup analysis forest plot for the length of ICU stay Source: References [9-12,16,20-25].

**Figure 9:**
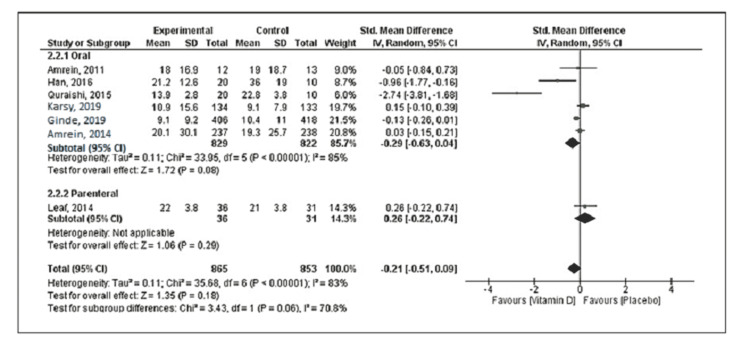
Subgroup analysis forest plot for the length of hospital stay Source: References [9-12,16,20-25].

Subgroup analysis was performed based on the route of administration (enteral or parenteral). More than 85% of patients were included in the enteral subgroup. The oral route of vitamin D administration was associated with the reduced length of ICU stay (SMD = -0.51; 95% CI = [-1.01, -0.00]; p = 0.05) without any difference in mortality, duration of mechanical ventilation, or length of hospital stay. Vitamin D supplementation via the parenteral route was associated with decreased risk of mortality (RR = 0.54; 95% CI = [0.35, 0.83]; p = 0.005), duration of mechanical ventilation (SMD = -0.77; 95% CI = [-0.1.26, -0.28]; p = 0.002), and length of ICU stay (SMD = -0.86; 95% CI = [-1.49, -0.22]; p = 0.008) without any difference in the length of hospital stay when compared to placebo (Figures 6-9). Table 2 shows sensitivity analysis by excluding each trial from the final analysis. Exclusion of the VIOLET trial shows a significant reduction in the risk of mortality in the vitamin D group compared to placebo.

**Table 2:** Sensitivity analyses after excluding individual studies RECTIFY: RandomizEd Clinical TrIal oF hYpovitaminosis D; VIOLET: Early high-dose vitamin D3 for critically ill vitamin D-deficient patients; VITdAL-ICU: Effect of high-dose vitamin D3 on hospital length of stay in critically ill patients with vitamin D deficiency; ICU: Intensive care unit; RR: Risk ratio; SMD: Standard mean difference. Source: References [9-12,16,20-25].

Trials	Mortality (RR)	Duration of mechanical ventilation (SMD)	Length of ICU stay (SMD)	Length of hospital stay (SMD)
Final Outcome	0.85 [0.68, 1.04]	-0.50 [-0.97, -0.03]	-0.60 [-1.03, -0.16]	-0.21 [-0.51, 0.09]
Trials excluded				
Amrein et al. (2011) [10]	0.82 [0.65, 1.03]	-0.57 [-1.11, -0.03]	-0.70 [-1.17, -0.24]	-0.23 [-0.56, 0.09]
Leaf et al. (2014) [21]	0.84 [0.67, 1.06]	NA	-0.43 [-0.81, -0.04]	-0.29 [-0.63, 0.04]
Amrein et al. (2014) [9]	0.82 [0.65, 1.03]	-0.66 [-1.07, -0.26]	-0.71 [-1.29, -0.13]	-0.35 [-0.77, 0.08]
Quraishi et al. (2015) [12]	0.83 [0.66, 1.05]	NA	-0.42 [-0.81, -0.03]	-0.02 [-0.20, 0.16]
Han et al. (2016) [11]	0.83 [0.66, 1.04]	-0.49 [-1.03, 0.04]	-0.60 [-1.07, -0.14]	-0.13 [-0.43, 0.18]
Miroliaee et al. (2017) [20]	0.90 [0.75, 1.08]	NA	NA	NA
Ding et al. (2017) [22]	0.85 [0.68, 1.06]	-0.43 [-0.94, 0.09]	-0.65 [-1.14, -0.16]	NA
Karsy et al. (2019) [25]	0.83 [0.66, 1.05]	NA	-0.72 [-1.27, -0.18]	-0.33 [-0.70, 0.05]
Hasanloei (2019) [23]	0.89 [0.73, 1.07]	-0.33 [-0.71, 0.05]	-0.54 [-1.00, -0.09]	NA
Miri et al. (2019) [24]	0.88 [0.71, 1.09]	-0.55 [-1.11, 0.01]	-0.61 [-1.09, -0.14]	NA
Ginde et al. (2019) [16]	0.77 [0.64, 0.93]	NA	NA	-0.32 [-0.77, 0.12]

Discussion

In this present meta-analysis of 2,187 patients from 11 RCTs, we evaluated the role of vitamin D supplementation in critically ill patients. The main findings are that vitamin D supplementation in critically ill patients was associated with reduced duration of mechanical ventilation and ICU stay. There was no significant difference noted in mortality and length of hospital stay. The parenteral route of vitamin D administration was associated with a reduction in the risk of mortality, duration of mechanical ventilation, and ICU stays as noted in the sub-group analysis of limited patients.

Increased incidence of vitamin D deficiency has been shown to be associated with critically ill patients with some studies reporting a prevalence close to 80% (1-3, 26-28). Vitamin D plays an important role in inflammatory pathways [4]. It inhibits various inflammatory cytokines such as IL-1 α, IL-1β, tumor necrosis factor (TNF)-α and affects T-lymphocyte differentiation by inhibiting IL-12 release from dendritic cells [29]. Decreased vitamin D levels are associated with a lower level of cathelicidin peptide that acts against infectious agents and has been associated with worse outcomes [30,31]. Several retrospective and prospective studies have reported that reduced levels of vitamin D were associated with increased mortality among critically ill patients [32-35]. Critically ill patients with sepsis, acute kidney injury, and other medical conditions have worse outcomes when they have concomitant vitamin D deficiency [32,36,37]. Increased rate of infection, ICU length of stay, duration of mechanical ventilation, and hospital stay along with increased health-care costs have been associated with vitamin D deficiency in critically ill patients [38,39].

So far, RCTs have been inconclusive regarding vitamin D supplementation in critically ill patients. Earlier trials had several limitations including variability among the trials in terms of dosages of vitamin D, route of administration, and the limited number of patients included. The VITdAL-ICU (vitamin D deficiency in critically ill patients) trial was one of the first large-scale trials, which looked at two different vitamin D deficiency groups (25(OH)D <20 ng/ml and <12 ng/ml). They found no overall difference in the six-month mortality but did find statistically significant mortality reduction in the subgroup analysis of the severely deficient vitamin D group (<12 ng/ml) [9]. However, the VIOLET trial failed to show any mortality benefits [19]. Important differences between the VITdAL-ICU and the VIOLET trial include the inclusion of patients early in the course of critical illness, using a vitamin D cutoff of <20 ng/ml and not providing additional vitamin D supplementation in the VIOLET trial. Our largest meta-analysis failed to show any mortality benefits, but unlike the VIOLET trial, we found a non-significant trend toward decreased mortality in the vitamin D group. Interestingly, sensitivity analysis after excluding the VIOLET trial shows a reduction in mortality with vitamin D supplementation. In our subgroup analysis, parenteral vitamin D supplementation showed significant mortality benefits; however, this subgroup involves less than 15% of the total study population, and none of the major trials (VITdAL-ICU or VIOLET) were included.

Our meta-analysis shows vitamin D supplementation reduces the duration of mechanical ventilation and ICU stay in critically ill patients, though the results are associated with significant heterogeneity. These results are important as both the long duration of mechanical ventilation and ICU stay are associated with increased long-term morbidity and mortality [40,41]. Additional sensitivity and subgroup analyses show a reduction in mechanical ventilation duration mainly in the parenteral subgroup, while the reduction in length of ICU stay is noted in both routes of vitamin D supplementation. The VIOLET trial was not included in these analyses as the length of ICU stay was not reported in the trial and only a small portion of patients in the trial had post-randomization mechanical ventilation.

When compared to previous meta-analyses [13-15], this updated analysis has some major differences. First, our study has a much larger population; second, many newer and more diverse clinical trials are included in this analysis, and finally, our results demonstrate benefits in terms of the duration of mechanical ventilation and ICU stay. Currently, two large-scale RCTs are ongoing, which will further improve the evidence regarding the role of vitamin D in critically ill patients [42,43].

## Conclusions

Vitamin D supplementation in critically ill patients decreases the duration of mechanical ventilation and the length of ICU stay. Vitamin D may reduce mortality in specific groups of critically ill patients. Our study has some important limitations. Despite including 11 trials, we still have a small population of patients in this study. There are many disparities among the trials in terms of dose and route of vitamin D supplementation, checking vitamin D levels in some trials, outcomes reported, and duration of follow-up. We included a very diverse population of critically ill patients like patients with neuro-critical illness, sepsis, ventilator-associated pneumonia, and trauma patients. All these differences led to significant heterogeneity among trials. Nevertheless, the results of this study are important and are hypothesis-generating regarding the duration of mechanical ventilation and ICU stay and the role of parenteral vitamin D supplementation. Further large-scale RCTs are needed to identify specific groups of critically ill patients who will most likely benefit from vitamin D supplementation.
